# Effects of Exergaming on College Students’ Situational Interest, Self-Efficacy, and Motion Sickness

**DOI:** 10.3390/jcm11051253

**Published:** 2022-02-25

**Authors:** Madeline R. Lawrence, Hung-I Wan, Wenxi Liu, Daniel J. McDonough, Shivani Mishra, Zan Gao

**Affiliations:** 1 College of Biological Sciences, University of Minnesota-Twin Cities, Minneapolis, MN 55455, USA; lawre427@umn.edu; 2 School of Kinesiology, University of Minnesota-Twin Cities, Minneapolis, MN 55455, USA; wan00046@umn.edu; 3 Department of Physical Education, Shanghai Jiao Tong University, Shanghai 200240, China; 4 School of Public Health, University of Minnesota-Twin Cities, Minneapolis, MN 55455, USA; mcdo0785@umn.edu; 5 Department of Population Health, NYU Grossman School of Medicine, New York, NY 10016, USA; shivani.mishra@nyulangone.org

**Keywords:** exergaming, situational interest, self-efficacy, motion sickness, college students

## Abstract

Objective: Given the low levels of physical activity (PA) among U.S. college students, the use of exergaming as a supplement to traditional exercise may promote higher levels of motivation and PA. Therefore, this study’s purpose was to examine the effect of two different exergames on college students’ situational interest (SI), self-efficacy (SE), and equilibrium change (EQC) compared to traditional treadmill walking. Methods: Sixty college students (30 female; *M_age_* = 23.6 ± 4.1 years; *M_BMI_* = 23.9 ± 4.0 kg/m^2^) participated in three separate 20 min exercise sessions: (1) Xbox 360 Kinect *Just Dance*; (2) Xbox 360 Kinect *Reflex Ridge*; and (3) traditional treadmill walking at 4.0 mph. Participants’ SI, SE, and EQC were measured after each session using a series of validated surveys. Results: A mixed model analysis of covariance (ANCOVA) with repeated measures evaluated mean differences between exercise sessions for all outcomes. Significant main effects were observed between the three exercise sessions (all *p* < 0.01). Specifically, *Just Dance* and *Reflex Ridge* sessions yielded significantly higher SI scores than treadmill exercise, F (10, 49) = 54.61, *p* < 0.01, η^2^ = 0.92. In addition, participants experienced significantly lower EQC in *Reflex Ridge* than in treadmill exercise, F (2, 58) = 4.26, *p* = 0.02, η^2^ = 0.13. No differences were identified for SE. Conclusion: The integration of exergaming into traditional exercise routines may help to promote higher levels of SI but not SE amongst college students. RR exergaming also demonstrated low EQC as compared to traditional exercise. Experimental study designs are warranted to provide additional evidence on the efficacy of exergaming.

## 1. Introduction

The prevalence of obesity has reached epidemic proportions in the United States with around 93.3 million adults affected, 35.7% of which are aged 20 to 35 [1]. Research has indicated obesity to be a leading risk factor for the development of chronic diseases, such as coronary heart disease, heart failure, and Type 2 diabetes [2,3], and has also shown to increase the risk of premature death later in life [4]. Indeed, only approximately 48% of college students meet the recommended guideline of ≥ 150 min of moderate-intensity or 75 min of vigorous-intensity physical activity (PA) set by the Centers for Disease Control and Prevention [5,6]. Low PA among college students is a multifaceted issue and may be the result of increased academic pressures and the newfound responsibility to balance school with work, social lives, and other responsibilities [7]. Given that the first few years of college are associated with weight gain and that preceding research has demonstrated physical inactivity (i.e., time awake spent sitting or lying down) to be an independent risk factor for obesity, college students are a population at high risk for developing obesity and other related health issues [8,9,10]. To encourage higher levels of PA in this population, novel and innovative PA intervention strategies should be explored. Exergaming is a type of video game that requires movement of the full body during gameplay where the game interacts with the player based on his or her movements [11]. Given that 65% of American adults play video games, exergaming represents one such strategy that can be used to promote PA [12]. Indeed, previous research has observed exergaming to promote PA in young adults.

One mechanism by which exergaming may promote increased PA in this population is by increasing PA-related situational interest (SI) and self- efficacy (SE). Indeed, previous studies indicate SI and SE to be positive predictors of emotional engagement, perseverance, and overall participation in PA settings [13,14]. In detail, SI refers to a momentary appealing effect of an activity on individuals in a particular context and at a particular moment, and SE is defined as an individual’s belief in his or her ability to learn and perform at a situational level, and it is positively correlated with higher PA engagement during exergaming [13,14]. One study examined the effects of exergaming on SE in young adults from a multidisciplinary perspective and found that exergaming may be a possible means to enhance PA in young adults due to a higher perception of enjoyment during the session as compared to traditional exercise on a treadmill [15]. Other studies also found increased feelings of vigor and lower overall rates of perceived exertion as compared to traditional exercise, supporting the idea that exergaming creates outcomes that enhance PA engagement [16,17,18].

Despite the promise of enhanced PA, studies examining the effect of exergaming on equilibrium change have also shown that traditional gaming can cause motion sickness (e.g., disoriented equilibrium) [19,20]. When playing traditional video games in either a standing or sitting position, adults report motion sickness at a rate as low as 42% and as high as 67% of the time [19,21]. Therefore, the possibility of equilibrium change (EQC) symptoms must be assessed when examining exergaming as a potential means of increasing PA levels.

This cross-sectional study was designed to examine the effect of the two exergames on EQC compared to traditional treadmill walking. We hypothesized that: (1) the two exergames would yield higher SI and SE scores compared to traditional treadmill exercise; and (2) the two exergames would result in a higher EQC compared to traditional treadmill exercise. The findings of this study will help establish effective PA promotion intervention strategies for college students, which may help combat the increased sedentary habits in this population.

## 2. Materials and Methods

### 2.1. Participants and Study Design

A total of 60 college students (30 females; 39 non-Hispanic white; M_age_ = 23.6 ± 4.1 years; M_BMI_ = 23.9 ± 4.0) were recruited for the study via flyers posted around the study University’s campus and by word of mouth. Participants completed a series of three separate 20-min exercise sessions in a random order during a single laboratory visit. The exercise sessions were: (1) Xbox 360 Kinect *Just Dance*, a game where players mirror the dance moves of an on-screen avatar in time with music (Microsoft, Redmond, WA, USA); (2) Xbox 360 Kinect *Reflex Ridge (RR)*, a game type on the Xbox 360 Kinect game Kinect Adventures, where players jump, lean, and duck to avoid obstacles; and (3) treadmill walking at 4.0 mph. Prior to data collection, the Institutional Review Board Approval (1502M62327) and written informed consent were obtained. Participants were compensated with a $20 ClinCard^®^ (Greenphire Inc., Philadelphia, PA, USA) upon successful completion of the study.

### 2.2. Measurements

#### 2.2.1. Anthropometric Measures

Trained research assistants measured height to the nearest half-centimeter using a Seca stadiometer (Seca, Chino, CA, USA), after which weight and body fat percentage were measured via bioelectrical impedance using the Tanita BC-558 IRONMAN^®^ Segmental Body Composition Monitor (Tanita, Tokyo, Japan) digital weight scale.

#### 2.2.2. Situational Interest (SI)

SI was measured using the adapted SI survey [22], which was completed immediately after the completion of each 20-min exercise session. The survey consisted of 14 questions based on the interest level elicited by the exercise to measure novelty (i.e., this activity is new to me), attention demand (i.e., my attention was high), instant enjoyment (i.e., it is an enjoyable activity to me), challenge (i.e., it is a complex activity), and exploration intention (i.e., I want to analyze it to have a grasp on it) for each exercise modality. A 5-point Likert scale, ranging from 1 = *very true* to 5 = *very untrue*, was used for all responses. The average scores of these 5 dimensions of SI were used as the outcomes. Notably, the lower score represents the higher level of each SI subscale.

#### 2.2.3. Self-Efficacy (SE)

SE was assessed using the self-efficacy survey, a 5-item assessment of participants’ perception of confidence in each exercise session, and was conducted on a 5-point Likert scale, with 1 = *strongly disagree* and 5 = *strongly agree* [23]. Sample questions included: “I have more fun playing exergames/traditional exercise than doing other things”; “Playing exergames/traditional exercise is the thing I like to do best”; and “I usually prefer to watch rather than play.” The average score of these 5 items was used to represent self-efficacy.

#### 2.2.4. Equilibrium Change (EQC)

EQC was measured using a survey with two questions to understand the equilibrium state post exercise [16]. The survey included these two statements: “I feel sick to my stomach” and “I feel dizzy”, and participants responded with one of these three answers: “not at all”, “a little bit”, and “a lot”. The average score of these 2 items was used as the outcome for EQC.

### 2.3. Procedures

After reading and signing the informed consent documents, participants reported demographic information including age, sex, and race/ethnicity. Height, weight, and body fat percentage measurements were also taken for each participant. Participants then completed three separate 20 min exercise sessions on Xbox 360 Kinect *Just Dance* in single-player “Competition” mode, Xbox 360 Kinect *Reflex Ridge* in single-player “Competition” mode, and treadmill walking in a randomly allocated order.

Given PA-related sex-norms [24], these two exergames were chosen to observe if males or females gravitated towards a particular type of exergame. The Sole F63 treadmill (Sole Fitness; Taipei, Taiwan) was used during the treadmill walking session, with the speed set at a consistent 4.0 miles per hour and a zero-degree incline. During the treadmill exercise, participants were allowed the option to either listen to music or watch videos using their phones/tablets. Participants completed SI, SE, and EQC evaluations immediately after the completion of each exercise. Ten minutes of rest were provided between each different exercise to allow the participants’ blood pressure to return to baseline and to limit a potential carryover effect from one session to the next [25].

### 2.4. Statistical Analyses

Statistical analyses were completed using SPSS 22.0 (IBM Inc.; Armonk, NY, USA). First, descriptive statistics (means and standard deviations (SD)) were calculated for all outcome variables, followed by a repeated measures (within-subjects factor: exercise session (3 levels)) analysis of covariance (ANCOVA) with gender (male vs. female) as the covariate to evaluate mean differences between exercise sessions for SI, SE, and EQC. The significance level was set at 0.05. Effect size for each comparison was measured by partial eta square (η^2^). In the present study, small, medium, and large effect sizes were designated as 0.01–0.06, 0.06 < 0.14, and > 0.14, respectively [26].

## 3. Results

Participants in this study were young adults with an average age of 23.6 years (SD = 4.1). The average height was 171.8 cm (SD = 8.2); the average weight was 70.7 kg (SD = 15.2); and the average BMI was 23.9 kg/m^2^ (SD = 4.0). Table 1 presents the descriptive statistics for SE, SI, and EQC across all three exercise sessions. Apart from EQC, the treadmill exercise sessions had lower scores for all outcomes compared to both exergames. Comparing the two exergames, *Just Dance* had higher scores than *Reflex Ridge* (RR) for all outcomes except EQC, which was higher in RR than in Just Dance.

Based on the ANCOVA analysis, significant main effects were revealed between the three exercise sessions (all *p* < 0.01). In detail, further analysis revealed significant main effects for SI (F (10, 49) = 54.61, *p* < 0.01, η^2^ = 0.92). Notably, the follow-up tests for SI revealed that there were no significant differences between Just Dance and RR for all five SI dimensions (novelty, attention demand, instant enjoyment, challenge, and exploration intention), but significant differences were observed between the two exergames and treadmill exercise. In particular, Just Dance and RR sessions yielded statistically significantly higher SI scores than treadmill exercise for all participants (*p* < 0.001). There were significant main effects for EQC (F (2, 58) = 4.26, *p* = 0.02, η^2^ = 0.13). Post hoc comparisons indicated that participants had significantly lower EQC during RR than during traditional treadmill exercise (*p* < 0.01). However, no significant main effects for SE were identified (*p* > 0.05). There was no significant effect for the covariate either.

## 4. Discussion

This study aimed to investigate and compare the effects of two exergames to traditional treadmill exercise on college students’ SI, SE, and EQC. The results indicated that participants had significant a lower EQC only in the *Reflex Ridge* exergaming session in comparison with traditional treadmill walking. Overall, the findings were in favor of exergaming with greater SI and lower EQC compared to traditional treadmill walking, which suggest the potential of implementing such exergaming programs among college students for promoting PA and well-being.

Exergaming is the combination of physical exercise and video games that caters to innovation and novel experiences by enhancing the participants’ attention and motivation; hence, enabling them to control and explore the dynamics of the game. The game provides unique experiences that allow the user to become the video’s main character and offers real-time movements [27,28,29,30,31,32]. The first hypothesis was partially met: participants in exergaming indicated high scores in SI but not in SE as compared to exercise on the treadmill. The hypothesis also agreed with a 4-week study examining children’s SI with exergaming sessions that recorded a high SI for exergaming compared to common physical exercises [17]. Intrinsic motivation is the ability of an individual to do something without expecting any external rewards [19]. Although uncommon among children, the exergaming process indicated otherwise.

The SI dimensions of attention demand and exploration opportunity depicted in the findings indicate that a higher cognitive ability is required when playing exergames compared to the traditional training sessions. The enhancement of cognitive abilities enables the students to create different mental models that assist them in decision-making processes and navigation through various obstacles while playing the game. This finding would enable the students to develop a broad understanding of physical activities and help them acquire knowledge of tactics and strategies in sports [20]. Playing video games engages the mind of the player and enhances their cognitive ability, hence promoting better and faster decision making [21,33]. This study’s findings are consistent with findings in previous studies on the role of cognitive demand for high SI and motivation, which showed that the cognitive demand of a learning task played a critical role in generating situational interest [34].

The SE theory states that the capability of engagement and accomplishment of a task, experience and social persuasion, contribute to SE in an individual [35,36,37]. In-built commentators in exergames such as Just Dance deliver comments like “perfect,” and “super,” which offer social persuasion that contributes to the SE of the participant. Even though exercising on a treadmill is close to taking a walk, or jogging outdoors, the treadmill lacks social persuasion that contributes to the SE of the students. According to a study performed on 160 undergraduates, the *Dance Dance Revolution* and Wii games yielded higher SE scores compared to a traditional 10 min treadmill walk [38]. However, the present study did not find significant differences between sessions on SE. As the prior exergaming experience was not assessed in this study, there may be an influence on the findings of SE during the exergaming sessions. Although just a video game, the exergames offer real-time and authentic experiences that are specific to each player.

Regarding the EQC, the findings were incongruent with the second hypothesis, which stated that both exergames would result in a higher EQC compared to traditional treadmill exercise. Previous research showed the prominence of EQC in the form of motion sickness during exercise sessions with immersive VR. Therefore, the low EQC observed could be a result of exergaming which did not require the participating students to wear head-mounted displays. Since most studies have been performed either on children or adults in a professional setting (i.e., flight pilot simulator training), very little literature is present on exergaming and its effects on young adults. Biological research also shows that the brain is the most neurologically plastic between the ages of 6 months and 25 years [39]. Symptoms of EQC (stomach sickness, dizziness, etc.) can be triggered due to a slow perceptual adaptation to an altered visual perception that is experienced during exergaming sessions using immersive VR. However, during this study, participants were observed to have lower EQC in exergaming compared to treadmill exercise. This may be explained by the lesser degree of immersion in the virtual world as compared to the fully immersive VR settings. On the other hand, this may be considered as a safer approach to promote PA among young adults who may be susceptible to motion sickness. In addition, the EQC was significantly higher on the traditional treadmill as compared to exergames which may be due to its physically demanding aspects [2]. Participants’ attention may be distracted during the interactive exergaming play, whereas participants may easily drive their attention in the physical perception as there is no interaction during the traditional exercise. In addition, the notion of movement without actually covering the distance creates a high equilibrium change which is also exhibited by Just Dance. “Attention Demand”, “Novelty”, and “Exploration Opportunity” are indicators that implied that SI could be enhanced through the motivational power of exergames.

The present study has several strengths, such as the comparison of psychological outcomes between two different exergames to traditional treadmill exercise, the sampling of a large population sparse in exergaming research, and the examination of psychosocial outcomes of the two exergames compared to the traditional treadmill exercise. However, there are some limitations which must be addressed. Since each participant only experienced a single 20 min trial, it is non-conclusive that SE and SI would sustain over longer periods. Future studies may adopt independent groups to further examine the difference between exergaming and traditional exercise. In addition, the participants’ prior exergaming experience was not assessed. Prior experience or exposure to the exergames may affect the outcomes of this study. For example, participants with prior exergaming experience may have higher SE compared to those who have not played before. Future studies need to pay attention to the participants’ characteristics and conduct baseline tests to avoid inconsistent outcomes. Lastly, because the nature of this research is a cross-sectional study, the findings cannot conclude a causal relationship between the SI, SE, and EQC of the students to the exergaming experience and traditional treadmill exercise. Therefore, there is a need for future studies to implement a randomized control design for further examination of the effects of exergaming on young adults’ PA and health-related outcomes. Future studies may also investigate the role of self-representational avatars on the players’ beliefs and motivation, such as SI and SE [40,41], during exergaming play.

## 5. Conclusions

Most sedentary video games are stereotyped to be mindless, violent, lacking in PA, and a contributing factor in the rise of obesity among the youth [20]. Exergaming as an innovative and fun active video game sheds light on motivating individuals to be physically active. The present study findings indicate that exergaming may increase SI and lower EQC compared to treadmill exercise in healthy college students. Taken together, it is feasible for colleges and stakeholders that are implementing exergaming stations on campuses to encourage and attract college students to engage in PA as opposed to living a relatively sedentary life and falling at risk of obesity and other physical inactivity-related diseases.

## Figures and Tables

**Table 1:** Descriptive characteristics of the outcome variables.

	Just Dance		Reflex Ridge		Treadmill	
	Mean	SD	Mean	SD	Mean	SD
Self-Efficacy	3.39	0.71	3.43	0.43	3.38	0.56
Attention Demand	1.65	0.50	1.76	0.57	2.98	0.66
Instant Enjoyment	2.25	0.57	2.29	0.60	3.24	0.59
Challenge	2.13	0.61	2.26	0.57	3.07	0.54
Novelty	1.91	0.59	1.90	0.63	3.37	0.48
Exploration Opportunity	1.75	0.49	1.81	0.52	3.39	0.54
Equilibrium Change	1.07	0.04	1.02	0.01	1.08	0.03

Note: SD, standard deviation.

## Data Availability

The data presented in this study are available on request from the corresponding author.
